# Comparison of enterovirus detection in cerebrospinal fluid with Bacterial Meningitis Score in children

**DOI:** 10.1590/S1679-45082017AO3880

**Published:** 2017

**Authors:** Frederico Ribeiro Pires, Andréia Christine Bonotto Farias Franco, Alfredo Elias Gilio, Eduardo Juan Troster

**Affiliations:** 1 Hospital Israelita Albert Einstein, São Paulo, SP, Brazil.; 2 Universidade de São Paulo, São Paulo, SP, Brazil.

**Keywords:** Meningitis/diagnosis, Meningitis, viral/diagnosis, Meningitis, bacterial/diagnosis, Enterovirus, Cerebrospinal fluid, Child

## Abstract

**Objective:**

To measure the role of enterovirus detection in cerebrospinal fluid compared with the Bacterial Meningitis Score in children with meningitis.

**Methods:**

A retrospective cohort based on analysis of medical records of pediatric patients diagnosed as meningitis, seen at a private and tertiary hospital in São Paulo, Brazil, between 2011 and 2014. Excluded were patients with critical illness, purpura, ventricular shunt or recent neurosurgery, immunosuppression, concomitant bacterial infection requiring parenteral antibiotic therapy, and those who received antibiotics 72 hours before lumbar puncture.

**Results:**

The study included 503 patients. Sixty-four patients were excluded and 94 were not submitted to all tests for analysis. Of the remaining 345 patients, 7 were in the Bacterial Meningitis Group and 338 in the Aseptic Meningitis Group. There was no statistical difference between the groups. In the Bacterial Meningitis Score analysis, of the 338 patients with possible aseptic meningitis (negative cultures), 121 of them had one or more points in the Bacterial Meningitis Score, with sensitivity of 100%, specificity of 64.2%, and negative predictive value of 100%. Of the 121 patients with positive Bacterial Meningitis Score, 71% (86 patients) had a positive enterovirus detection in cerebrospinal fluid.

**Conclusion:**

Enterovirus detection in cerebrospinal fluid was effective to differentiate bacterial from viral meningitis. When the test was analyzed together with the Bacterial Meningitis Score, specificity was higher when compared to Bacterial Meningitis Score alone.

## RESUMO

**Objetivo:**

Avaliar o papel da pesquisa de enterovírus no líquido cefalorraquidiano em comparação com o Escore de Meningite Bacteriana em crianças com meningite.

**Métodos:**

Coorte retrospectiva, realizada pela análise de prontuários, incluindo pacientes pediátricos, com diagnóstico de meningite e atendidos em um hospital privado e terciário, localizado em São Paulo, entre 2011 e 2014. Foram excluídos os pacientes com doença crítica, púrpura, derivação ventricular ou neurocirurgia recente, imunossupressão, outra infecção bacteriana concomitante que necessitasse de antibioticoterapia parenteral e aqueles que receberam antibiótico 72 horas antes da punção lombar.

**Resultados:**

Foram incluídos no estudo 503 pacientes. Destes, 64 foram excluídos e 94 não realizaram todos os exames para análise. Dos 345 pacientes restantes, 7 ficaram no Grupo de Meningite Bacteriana e 338 no Grupo de Meningite Asséptica. Não houve diferença estatística entre os grupos. Na análise do Escore de Meningite Bacteriana, dos 338 pacientes com possível meningite asséptica (culturas negativas), 121 deles tiveram um ou mais pontos para o Escore de Meningite Bacteriana, com valor de sensibilidade de 100%, especificidade de 64,2% e valor preditivo negativo de 100%. Dos 121 pacientes com Escore de Meningite Bacteriana positivo, 71% (86 pacientes) tiveram a pesquisa de enterovírus positiva no líquido cefalorraquidiano.

**Conclusão:**

A pesquisa de enterovírus no líquido cefalorraquidiano mostrou-se eficaz em diferenciar a meningite bacteriana da viral. Analisada junto com o Escore de Meningite Bacteriana, a especificidade foi maior em comparação ao Escore de Meningite Bacteriana isolado.

## INTRODUCTION

Meningitis is an inflammatory disease of the meninges, the tissue surrounding the brain and spinal cord, defined by changes in the cerebrospinal fluid (CSF), especially in the number of abnormal leukocytes. Acute meningitis includes bacterial meningitis and aseptic meningitis.

In bacterial meningitis, blood and/or CSF cultures are positive for routine bacterial pathogens. It is a life threatening condition, with mortality rates near 100% when not treated properly, requiring immediate treatment with empirical intravenous antibiotic therapy and life support management.^1^

In regions of the world with high vaccination rates, the incidence of bacterial meningitis has decreased substantially due to the high effectiveness of conjugate vaccines, especially against Type B *Haemophilus influenzae* and *Streptococcus pneumoniae.* ^2-8^

Aseptic meningitis may have infectious and non-infectious causes. The most common cause is enterovirus infection,^9^ a benign, self-limited condition that can be treated symptomatically on an outpatient basis.^1^

The majority of patients with aseptic meningitis are unnecessarily hospitalized and receive empirical intravenous antibiotics until the results of blood and CSF cultures are available,^10,11^ which may take 48 hours to rule out an infection caused by microorganisms.^12-14^ An epidemiologic study of meningitis in the United Kingdom concluded that it is urgent to improve the diagnosis of non-bacterial meningitis to reduce the use of antibiotics and admission to hospital.^8^

Taking into account that hospitalizations should be reduced as well as the unnecessary use of antibiotics, Nigrovic et al., proposed a score for the diagnosis of bacterial meningitis, called Bacterial Meningitis Score (BMS),^15^ which has a sensitivity and a negative predictive value close to 100%.

The BMS score has already been used in Brazil. It takes into account one clinical criterion (presence of seizures), and four laboratory criteria (positive CSF Gram stain, CSF absolute neutrophil count >1,000/mm^3^, blood absolute neutrophil count >10,000/mm^3^, and CSF protein >80mg/dL). The score has high sensitivity for bacterial meningitis, when one or more of the five criteria are present, and in differentiating from aseptic meningitis, but low specificity.

Enterovirus infection is the most common cause of aseptic meningitis in children and adults, and may cause up to 90% of cases of aseptic meningitis.^16^ Fast and accurate diagnosis of enterovirus infections can reduce the use of antibiotics, the length of hospital stay, and the financial costs of treating children with meningitis.^17-20^ With this in mind, several studies have been conducted to demonstrate the effectiveness of enterovirus detection in the diagnosis of aseptic meningitis.^21,22^

Molecular methods for virus testing in CSF are increasingly available. Therefore, the raised hypothesis is that using CSF enterovirus detection associated with the BMS could increased specificity of the diagnosis, keeping the sensitivity high, and thus reducing some unnecessary hospitalizations.

## OBJECTIVE

To evaluate the role of cerebrospinal fluid enterovirus detection compared to the *Bacterial Meningitis Score* in children with meningitis.

## METHODS

This analytical retrospective cohort study was conducted from April 2015 to November 2015, based on a review of medical records. It included patients aged 1 month to <14 years, diagnosed with meningitis (leukocytes in CSF >9cells/μL, taking into account a leukocyte: erythrocyte correction rate of 1:500 in case of puncture accident), and treated at the Emergency Department of *Hospital Israelita Albert Einstein*, located in the State of Sao Paulo, Brazil, from January 1st, 2011 to December 31st, 2014. Patients with critical illness (defined as having severe mental status alteration, evidence of cerebral herniation, need for respiratory or blood pressure support), purpura, recent ventricular shunt placement or other neurosurgery, immunosuppression, concomitant bacterial infection requiring parenteral antibiotics, or who received antibiotics 72 hours before lumbar puncture, were excluded.

The variables studied were age, sex, presence or history of seizures during the current condition, serum neutrophil count (1,800-10,000/mm^3^), CSF neutrophil count (up to 9/mm^3^), CSF protein (up to 40mg/dL), CSF Gram stain (positive/negative), blood culture (positive/negative), CSF culture (positive/negative), BMS (positive when at least one of the five criteria was present, and negative in the absence of all of them), and CSF enterovirus detection (positive/negative). Patients who had positive CSF bacterial cultures or pleocytosis (>9/mm^3^) associated with positive blood cultures for the bacterial pathogen were considered with bacterial meningitis. The data were collected by the researchers.

The sex of patients was described by the absolute frequencies and percentages per group, and compared by the Fisher’s exact test. Numerical variables were described as medians and quartiles, and compared by the Mann-Whitney test. To evaluate the BMS in differentiating patients with bacterial and aseptic meningitis, we calculated the measures of sensitivity, specificity, positive predictive value, negative predictive value and accuracy, and the Kappa coefficient, all measures with their 95% confidence intervals (95%CI). Calculations were made according to Altman’s recommendations^23^ and using the R version 3.1.3 (http://www.R-project.org) and Microsoft Excel version 2010. The level of significance adopted in the comparisons was 5%.

The sample size estimation was based on the specificity observed by Mekitarian Filho et al.,^7^ since a sensitivity of 100% was also required for this study. Assuming BMS has a 53% accuracy in the identification of children with aseptic meningitis (specificity), and an absolute accuracy of 5%, a sample of 383 cases of meningitis was estimated.

The formula for calculating the sample size was used to estimate a proportion with a significance level of 5%. Assuming that the number of cases fulfilling the inclusion criteria in the service totaled about 100 per year, we defined a 5-year period for the cohort to ensure the inclusion of the minimum number of cases required.

## RESULTS

**Figure 1:**
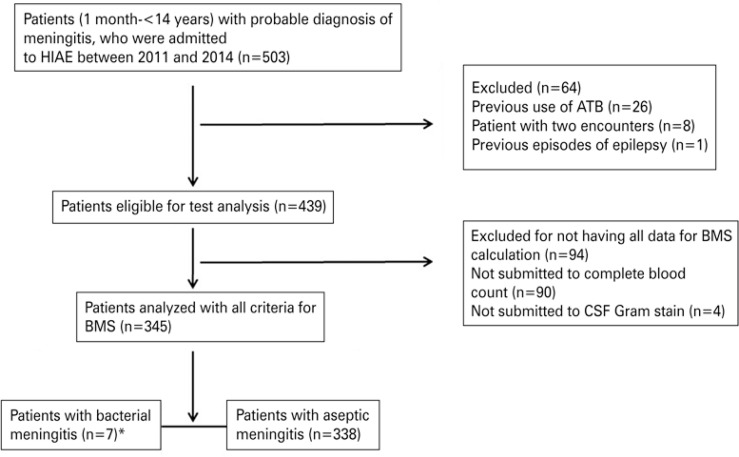
Flowchart

A total of 503 patients were included in the study; they had been admitted to *Hospital Israelita Albert Einstein* between 2011 and 2014 met the inclusion criteria. Of these, 64 were excluded due to previous use of antibiotics (n=26), no diagnosis of meningitis (n=29), patients with two emergency room admissions (n=8), and prior episode of epilepsy (n=1). There were 439 children eligible for medical record review. Ninety-four of them did not undergo all tests for analysis of BMS (90 were not submitted to complete blood count and 4 to CSF Gram stain), and were excluded from the study. Therefore, 345 patients met all requirements and were selected for the study - seven were in the Bacterial Meningitis Group, and 338 in the Aseptic Meningitis Group (Figure 1).

*5 cases caused by Streptococcus pneumonia; 1 case caused by *Neisseria meningitides*; 1 case caused by *Enterococcus faecalis*.

ATB: antibiotics; BMS: Bacterial Meningitis Score; CSF: cerebrospinal fluid.

When comparing the two groups, there was no statistical difference with respect to median age, sex, and blood neutrophil count (Table 1). The Bacterial Meningitis Group, in comparison to the Aseptic Meningitis Group, had greater CSF neutrophil count (1866/mm^3^ *versus* 32.5/mm^3^), blood polymerase chain reaction (23.3mg/dL *versus* 1.53mg/dL), and CSF protein (83mg/dL *versus* 30mg/dL) − all measures with statistically significant values shown in table 1.

**Table 1:** Parameters used to compare the groups

Comparative data	Aseptic meningitis (n=338)	Bacterial meningitis (n=7)	p value
Sex (%)			
Female	138 (40.8)	2 (28.6)	0.705
Male	200 (59.2)	5 (71.4)	
Age, median [IQR]	5.08 [3.58-7.08]	3.17 [0.67-6.25]	0.266
Neutrophils in CSF*, median [IQR]	32.50 [10.00-94.25]	1,866.00 [939.00-2,573.00]	<0.001
Neutrophils in blood^†^, median [IQR]	8,524.00 [6,065.00-11,086.75]	18,144.00 [8,293.50-24,173.00]	0.066
Protein in CSF, median [IQR]^‡^	30.00 [23.00-40.75]	83.00 [73.00-275.00]	<0.001

* neutrophils in CSF/mm^3^; ^†^ neutrophils in blood/mm^3^; ^‡^ protein in CSF (mg/dL). IQR: interquartile range; CSF: cerebrospinal fluid.

**Figure 2:**
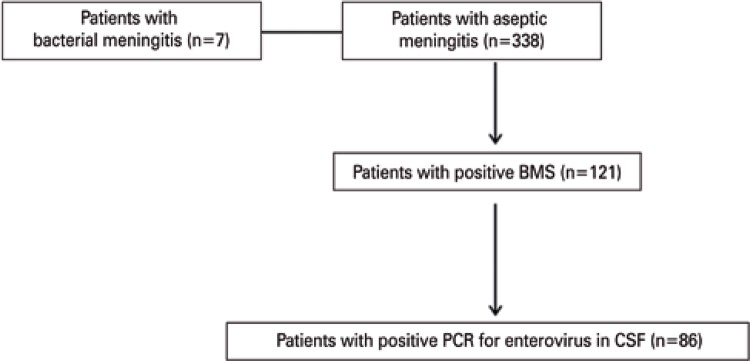
Impact of enterovirus detection

BMS: Bacterial Meningitis Score; PCR: polymerase chain reaction; CSF: cerebrospinal fluid.

A total of 345 patients were evaluated by BMS analysis. Of the 338 patients with aseptic meningitis (negative cultures), 121 had one or more BMS points (Table 2), with a sensitivity of 100%, specificity of 64.2%, negative predictive value of 100%, and kappa coefficient of 0.07 (Table 3). Of the 121 patients with positive BMS, 71% (86 patients) had positive CSF enterovirus detection (Figure 2).

**Table 2:** Correlation between the Bacterial Meningitis Score with at least 1 point and diagnosis of bacterial meningitis

BMS present	Bacterial meningitis		Total

	Yes	No	
Yes	7	121	128
No	0	217	217

Total	7	338	345

BMS: Bacterial Meningitis Score.

**Table 3:** Sensitivity, specificity, positive predictive value and negative predictive value of the Bacterial Meningitis Score for diagnosis of bacterial meningitis

Variables		Confidence intervals	
	
	Values n (%)	Lower limit 95%CI	Upper limit 95%CI
Sensitivity	100.0	100.0	100.0
Specificity	64.2	64.1	64.3
Accuracy	64.9	64.8	65.1
Prevalence	2.0	2.0	2.0
Positive predictive value	5.5	5.4	5.5
Negative predictive value	100.0	100.0	100.0
Kappa coefficient	0.07	-0.07	0.20

95%CI: 95% confidence interval.

Bacterial Meningitis Score and enterovirus detection combined, considering a probable bacterial meningitis (positive BMS + negative enterovirus detection), or a probable aseptic meningitis (positive enterovirus detection independent of the BMS result, or a negative BMS + negative enterovirus detection), yielded a sensitivity of 100%; specificity of 89.6%; prevalence of 2%; negative predictive value of 100%; Kappa coefficient of 0.26 (Tables 4 and 5).

**Table 4:** Correlation between Bacterial Meningitis Score and enterovirus detection with diagnosis of bacterial meningitis

BMS + enterovirus	Bacterial meningitis		Total

	Yes	No	
Positive BMS + negative enterovirus	7	35	42
Positive enterovirus + positive or negative BMS/negative BMS + negative enterovirus	0	303	303

Total	7	338	345

BMS: Bacterial Meningitis Score.

**Table 5:** Sensivity, specificity, positive predictive value and negative predictive value of Bacterial Meningitis Score associated to enterovirus detection for diagnosis of bacterial meningitis

Variables		Confidence intervals	
	
	Values n (%)	Lower limit 95%CI	Upper limit 95%CI
Sensitivity	100.0	100.0	100.0
Specificity	89.6	89.6	89.7
Accuracy	89.9	89.8	89.9
Prevalence	2.0	2.0	2.0
Positive predictive value	16.7	16.0	17.3

95%CI: 95% confidence interval.

## DISCUSSION

Several studies are underway to help differentiating bacterial from aseptic meningitis, in order to reduce hospitalization rates and therapeutic costs in aseptic meningitis cases, without decreasing sensitivity to bacterial meningitis. The first score was created by a retrospective cohort study^15^ published in 2002, conducted at the Boston Children’s Hospital, which evaluated 696 children aged between 1 month and 19 years, from July 1992 to June 2000, and found bacterial meningitis in 125 children, and aseptic meningitis in 571 children. The score had the following results: BMS=1 point with sensitivity of 100% (95%CI: 98 to 100%), and BMS=2 points with sensitivity of 87% (95%CI: 72 to 96%).

All over the world, some studies followed the same line to analyze BMS in their countries. In 2012, a meta-analysis^2^ with eight studies from Western Europe, United States and Argentina evaluated the BMS. The results showed a sensitivity of 99.3% (95%CI: 98.7-99.7), a specificity of 62.1% (95%CI: 60.5-63.7), a negative predictive value of 99.6% (95%CI: 99.3-99.8), and a positive predictive value of 28.1% (95%CI: 22.6-33.9). This shows a good sensitivity to bacterial meningitis, but the low specificity raises the number of unnecessary hospitalizations.

In Brazil, Mekitarian Filho et al.,^7^ also found excellent sensitivity using BMS in children with meningitis treated at the *Hospital Universitário da Universidade de São Paulo*, São Paulo (SP), observing a sensitivity and a negative predictive value of 100% with the score. However, the number of Brazilian and international studies conducted in private hospitals is still scarce, and this may generate controversy over the data, due to the fact that, at private services, more exams are requested and the patients seek treatment soon.

In this study, we evaluated the BMS of 1 point in 345 patients at a private hospital in São Paulo and obtained a sensitivity of 100% and a specificity of 64.2%, which are consistent with the results of other studies, but with a Kappa index of low reliability (0.07), most probably due to the low prevalence of bacterial meningitis (2%). This low prevalence is due to vaccination, and is similar to that observed in developed countries, where vaccination drastically reduced the incidence of bacterial meningitis.^2-8^

In the same line of reasoning to differentiate aseptic from bacterial meningitis, there are studies^21,24-27^ evaluating enterovirus detection in CSF, since enterovirus infection is the leading cause of aseptic meningitis. Enterovirus detection is usually done by polymerase chain reaction (PCR), which identifies viral RNA, or by virus culture. More recently, it has been possible to detect it by the genexpert enterovirus assay (GXEA) technique, which has been studied and compared with the gold standard technique.^1,9^

A large multicenter study showed no bacterial co-infection in 735 children with documented PCR for enterovirus,^28^ raising the certainty that a positive enterovirus detection rules out the diagnosis of bacterial meningitis. Therefore, fast and accurate detection tests documenting a viral etiology have a great potential of impact on the clinical management of aseptic meningitis and to reduce costs.^29^

The potential impact of PCR enterovirus detection on the management of aseptic meningitis was illustrated in pediatric studies,^21-27,30^ showing that detection of enteroviruses was associated with reduced length of hospital stay^21-26,30^ and shorter empirical antibiotic therapy.^21,24^ Some studies also indicated a decrease in hospitalization costs.^24,25^

No study in the literature evaluated BMS and enterovirus detection combined, or compared them. Of the 121 patients with BMS of 1 point or more, 86 had enterovirus detected, which could reduce admissions to hospital by up to 71%, since enterovirus patients do not require intravenous antibiotic therapy.

Enterovirus detection is not very widespread in Brazil, even in private services, because it is an expensive test. However, evaluating the probable benefit of reducing hospitalizations, the cost of the test would probably be beneficial. The use of enterovirus detection would not reduce admissions completely, since some cases of enterovirus meningitis requiring hospitalization to control symptoms, such as vomiting and headache, could still occur; however it would reduce hospitalizations for empirical antibiotic treatment, which usually last at least 48 hours until culture results are available.

Combining the score with detection yields a sensitivity of 100% and a specificity higher than when detection alone is used, with a value of 89.6%. The Kappa coefficient does not show good reliability, with a value of 0.26, but it points to the need of conducting new studies with a larger sample of patients.

## CONCLUSION

Cerebrospinal fluid enterovirus detection proved to be effective in differentiating bacterial meningitis from viral meningitis, leading to a probable reduction in hospitalization rates, unnecessary use of antibiotics, and costs. Combined with the Bacterial Meningitis Score, it yielded higher specificity than when the Bacterial Meningitis Score alone was used.
